# Repurposing Benzimidazoles against Causative Agents of Chromoblastomycosis: Albendazole Has Superior In Vitro Activity Than Mebendazole and Thiabendazole

**DOI:** 10.3390/jof9070753

**Published:** 2023-07-16

**Authors:** Rowena Alves Coelho, Maria Helena Galdino Figueiredo-Carvalho, Fernando Almeida-Silva, Vanessa Brito de Souza Rabello, Gabriela Rodrigues de Souza, Leandro Stefano Sangenito, Luna Sobrino Joffe, André Luis Souza dos Santos, Maria Cristina da Silva Lourenço, Marcio L. Rodrigues, Rodrigo Almeida-Paes

**Affiliations:** 1 Laboratório de Micologia, Instituto Nacional de Infectologia Evandro Chagas, INI/Fiocruz, Rio de Janeiro 21040-900, RJ, Brazil; maria.helena@ini.fiocruz.br (M.H.G.F.-C.); fernando.almeida@ini.fiocruz.br (F.A.-S.); vanessa.brito@ini.fiocruz.br (V.B.d.S.R.); 2 Plataforma de Bioensaios RPT 11B, Instituto Nacional de Infectologia Evandro Chagas, INI/Fiocruz, Rio de Janeiro 21040-900, RJ, Brazil; gabirspharma@hotmail.com (G.R.d.S.); cristina.lourenco@ini.fiocruz.br (M.C.d.S.L.); 3 Laboratório de Estudos Avançados de Microrganismos Emergentes e Resistentes, Departamento de Microbiologia Geral, Instituto de Microbiologia Paulo de Goés, Universidade Federal do Rio de Janeiro, Rio de Janeiro 21941-902, RJ, Brazil; ibastefano@hotmail.com (L.S.S.); andre@micro.ufrj.br (A.L.S.d.S.); 4 Instituto Federal de Educação, Ciência e Tecnologia do Rio de Janeiro, Nilópolis 26530-060, RJ, Brazil; 5 Department of Microbiology and Immunology, Stony Brook University, Stony Brook, NY 11792, USA; lujoffe@gmail.com; 6 Rede Micologia RJ, Fundação de Amparo à Pesquisa do Estado do Rio de Janeiro (FAPERJ), Rio de Janeiro 21941-901, RJ, Brazil; 7 Instituto Carlos Chagas, Fundação Oswaldo Cruz, Curitiba 81350-010, PR, Brazil; marcio.rodrigues@fiocruz.br; 8 Instituto de Microbiologia, Universidade Federal do Rio de Janeiro (UFRJ), Rio de Janeiro 21941-902, RJ, Brazil

**Keywords:** chromoblastomycosis, *Fonsecaea pedrosoi*, drug repositioning, benzimidazoles, albendazole

## Abstract

Chromoblastomycosis (CBM) is a neglected human implantation mycosis caused by several dematiaceous fungal species. Currently available therapy is usually associated with physical methods, especially surgery, and with high refractoriness. Therefore, drug discovery for CBM is essential. Drug repositioning is a strategy used to facilitate the discovery of new treatments for several diseases. The aim of this study was to discover substances with antifungal activity against CBM agents from a collection of drugs previously approved for use in human diseases. A screening was performed with the NIH Clinical Collection against *Fonsecaea pedrosoi*. Ten substances, with clinical applicability in CBM, inhibited fungal growth by at least 60%. The minimum inhibitory concentration (MIC) of these substances was determined against other CBM agents, and the benzimidazoles albendazole, mebendazole and thiabendazole presented the lowest MIC values. The selectivity index, based on MIC and cytotoxicity of these substances, revealed albendazole to be more selective. To investigate a possible synergism of this benzimidazole with itraconazole and terbinafine, the chequerboard method was used. All interactions were classified as indifferent. Our current results suggest that benzimidazoles have repositioning potential against CBM agents. Albendazole seems to be the most promising, since it presented the highest selectivity against all dematiaceous fungi tested.

## 1. Introduction

Chromoblastomycosis (CBM) is an implantation mycosis caused by several dematiaceous fungal species widely found in nature, which are endemic in rural areas of tropical and subtropical regions. CBM is an important public health problem, affecting poor populations in areas with limited access to medications and medical care [1,2], factors that led the World Health Organization (WHO) to classify CBM as a neglected tropical disease. In addition, the therapy currently available, based mainly on the administration of itraconazole and/or terbinafine, has numerous disadvantages, such as long duration and high cost, which are usually associated with physical methods, especially surgical removal of lesions. These approaches, however, present high relapse rates [2]. The discovery of new effective drugs against the agents of this neglected disease is therefore essential.

The process to develop a new drug is long and complex [3,4]. The slow pace of antifungal drug development, especially for neglected mycoses, is due to a variety of factors: the lack of interest from large pharmaceutical companies in antimicrobials [5]; the sharing of many biological pathways between humans and fungi [6]; and the difficulty and high costs to conduct clinical trials with adequate power [7]. Furthermore, the development of new antifungal drugs lags considerably behind when compared to other therapeutic areas. For example, from 2000 to 2015, 18 drugs were approved by the Food and Drug Administration for use in solid tumor cancers [8]. Meanwhile, six antifungal drugs were approved: caspofungin, micafungin, anidulafungin, efinaconazole, tavaborole, and isavuconazole [9,10,11,12,13]. More recently, ibrexafungerp and oteseconazole were also approved in 2021 and 2022, respectively [14,15]. Ibrexafungerp and isavuconazole are active against *Fonsecaea pedrosoi*, the main CBM agent [16,17], but for the other new approved antifungal drugs (2000–2022), there are no studies on CBM agents or the studies showed no satisfactory activity [18,19,20].

Drug repositioning is a strategy that has been used to facilitate the discovery and introduction of drugs for the treatment of several diseases caused by bacteria, protozoa, and fungi [21,22,23]. Nowadays, drug repurposing and screening of chemical libraries of synthesized compounds are prominent and became a priority in drug discovery programs, both in the industrial and academic sectors [4,24,25,26]. In the antifungal development area, sertraline, an antidepressant agent, has been reported as an in vitro and in vivo fungicidal compound that, in combination with amphotericin B, improves the outcome of cryptococcosis treatment [27,28]. According to Zhang et al. [29], there are several drugs demonstrating antifungal activity in preclinical studies, among them, antitumor agents, such as miltefosine [30], tamoxifen [31], methotrexate [32], and antiepileptic drugs [33].

The aim of this study was to find substances with antifungal activity against some CBM agents in a collection of drugs previously approved for use in other human diseases, thus suggesting this drug, with broad antifungal activity against the CBM agents, to be repositioned and tested in future clinical trials for the treatment of this neglected disease.

## 2. Materials and Methods

### 2.1. Strains and Growth Conditions

In this study, eight fungal isolates obtained from the Fiocruz Pathogenic Fungi Collection (CFP) and the American Type Culture Collection (ATCC^®^) were used. *Fonsecaea pedrosoi* (CFP 00791) was used throughout the study. This species is one of the main CBM agents worldwide [34]. *Cladophialophora carrionii* (CFP 00910), *Phialophora verrucosa* (CFP 00937), *Fonsecaea monophora* (CFP 00911), *Fonsecaea nubica* (CFP 00912), *Rhinocladiela similis* (CFP 00790), *Exophiala heteromorpha* (ATCC^®^ 28180), and *Exophiala dermatitidis* (ATCC^®^ 28869) were used to determine the minimum inhibitory concentration (MIC) and in synergism assays. Strains were maintained on potato dextrose agar (PDA) (Sigma Chemical Corporation, St. Louis, MO, USA). Seven-day cultures incubated at 30 °C were used in the assays.

### 2.2. Screening for Antifungal Activity in a Collection of Substances

The National Institute of Health (NIH) clinical collection (NCC) was screened for antifungal activity against the *F. pedrosoi* strain (CFP 00791). The NCC consists of a repository of molecules containing 727 substances arranged in 96-well plates distributed at 10 mM in dimethylsulfoxide (DMSO). These substances are part of the NIH Roadmap Molecular Libraries Screening Centers Network screening library, a collection of chemically diverse substances in Phase I-III clinical trials [35]. Each substance was initially diluted to 1 mM in DMSO and stored at −20 °C until use. For the initial screening, all substances were used at 10 μM in 100 μL of RPMI 1640 medium (twice concentrated) buffered with morpholinepropanesulfonic acid (MOPS) at a final concentration of 0.165 mol/L pH 7, containing 2% glucose, in 96-well plates. The final DMSO concentration corresponded to 0.5% *v*/*v*. The inoculum was prepared according to the protocol for filamentous fungi proposed by the European Committee on Antimicrobial Susceptibility Testing—EUCAST, with minor modifications [36]. The *F. pedrosoi* isolate (CFP 00791) was grown on PDA medium and incubated at 35 °C for seven days. After growth, conidia were suspended in 3 mL of sterile distilled water with 0.1% Tween 20, vortexed, and the concentration adjusted to the 0.5 McFarland scale. The suspension was then diluted 1:10 in order to obtain a final working inoculum at 2–5 × 10^5^ CFU/mL. Next, 100 µL of the inoculum was added to each well and the plates incubated at 35 °C for 72–96 h. Negative and positive controls consisted of culture medium alone and drug-free culture medium with the fungal inoculum previously described, respectively. Plates were read using a microplate reader (Epoch, Biotek, Wenusky, VT, USA) with a 530 nm filter to obtain the optical density. All plates were also visually read to confirm the equipment data. A calculation was performed to obtain the percentage of inhibition of fungal growth, according to the formula: (1 − (OD1/OD2)) × 100, where OD1 = optical density of the fungus in the presence of the drug; and OD2 = optical density of the growth control drug-free well. Drugs that showed growth inhibition values greater than 60% (hereafter named hit drugs) in at least two independent experiments were selected for further studies. The DrugBank platform (https://go.drugbank.com/ accessed on 20 May 2021) was used to search for information on routes of administration and adverse effects of selected substances.

### 2.3. Determination of the Minimum Inhibitory Concentration of Hit Drugs

Minimum inhibitory concentrations (MICs) were determined using the method proposed by the European Committee on Antimicrobial Susceptibility Testing (EUCAST), with minor modifications. Hit drugs were serially diluted (80 μM to 0.15625 μM) in RPMI 1640 (twice concentrated, pH 7; supplemented with 2% glucose) buffered with MOPS in 96-well plates. The inoculums of all strains were prepared following the EUCAST protocol [36]. Plates were incubated at 35 °C for 72–96 h. The MIC of the NCC substances was determined as the lowest concentration capable of inhibiting 100% of fungal growth when compared to the growth control well. MIC values were determined in duplicate.

### 2.4. Determination of the Minimum Fungicidal Concentration of Hit Drugs

Minimum fungicidal concentration (MFC) was determined in triplicate by transferring a 5 μL aliquot from each well without fungal growth of the microdilution plates used for MIC determination, as described above, onto 2% glucose Sabouraud agar (Sigma Chemical Corporation). The MFC was determined as the lowest drug concentration without fungal growth on Sabouraud agar after five days of incubation at 35 °C. When the MFC/MIC ratio of a hit drug was 1 or 2, the substance was considered fungicidal against the pathogen. If this ratio was greater than 2, the hit drug was considered to be fungistatic [37,38].

### 2.5. Synergism of Hit Drugs with Itraconazole and Terbinafine

To assess the type of interaction between the hit drugs and the antifungal drugs most used in the treatment of CBM (itraconazole and terbinafine), the fractional inhibitory concentration index (FICI) was determined, which defines the type of interaction between drugs in combination as follows: synergism if FICI ≤ 0.5; indifference if 0.5 < FICI ≤ 4; and antagonism if FICI > 4 [39]. The FICI was obtained by the sum of the fractional inhibitory concentrations (FIC) or by the formula: (A/MIC(a) = FIC_A) + (B/MIC(b) = FIC_B) = FICI, where: A = MIC of the drug (a) in combination; MIC (a) = MIC of drug (a) alone; B = MIC of drug (b) in combination; and MIC (b) = MIC of drug (b) alone [40,41]. The test was carried out using the chequerboard method, in which the two drugs were applied in a single well of a 96-well plate, so that in each of the wells there were different concentrations of the combinations between the substance and the antifungal agent (Figure S1). Drug dilutions were prepared following the methodology proposed by EUCAST, starting from a stock solution of the hit drug or antifungal drug 100× concentrated, according to the methodology described to determine the MIC [36]. Hit drug A/antifungal drug B serial microdilution preparations were performed following the above mentioned protocol, observing the final concentration in the wells after the addition of drugs and inoculum. Briefly, 50 μL of each drug to be combined were distributed, which resulted in a final volume of 100 μL in each microplate well, with the exception of the first column reserved for culture medium control (without the fungal inoculum) and the last well on the right without drugs, reserved for fungal growth control. Column 12 was reserved for drug B (itraconazole or terbinafine) susceptibility testing. Row H was reserved for the selected NCC substance. The other combined drug and substance concentrations were distributed between columns 2 and 11 in rows A to G. Drug concentrations ranged from 0.062 μM to 4 µM for itraconazole and terbinafine, and from 0.125 μM to 64 µM for the hit drug on the combination boards.

### 2.6. Evaluation of Cytotoxicity and Determination of the Selectivity Index (SI)

VERO cells (ATCC^®^ CCL-81, a kidney tissue derived from a normal adult African green monkey) were cultured in 199 medium with Earle’s salts supplemented with 100 U/mL of penicillin–100 µg/mL of streptomycin (Cultilab LTDA, São Paulo, Brazil) and 10% fetal bovine serum (FBS, Cultilab LTDA, Brazil) in an incubator at 37 °C with 5% CO_2_. Cells were subcultured in 25 or 75 cm^2^ culture flasks once a week and the culture medium was also changed once a week. The cells used in the experiments were from passages 10 to 29 [42]. Cytotoxicity assays to assess cell viability were performed in 96-well plates with 5 × 10^4^ cells/well that were exposed to hit drug solutions for 24 h at 37 °C in an incubator with 5% CO_2_. Initially, cells were treated with a range of concentrations of hit drugs (3125–100 µg/mL) to determine the CC_50_ (concentration that inhibits 50% of growth). A solution of 10% Tween 80 solution was used as the positive cellular death control [43]. Wells with VERO cells receiving only the culture medium with or without 1% DMSO were also included as positive cellular growth controls.

The 3-(4,5-dimethylthiazol-2-yl)-2,5-diphenyltetrazolium bromide (MTT, Sigma-Aldrich^®^, USA) reagent dissolved in PBS, pH 7.4, was used to check cell viability. Results were read at 492 nm on a Thermo Scientific^®^ Multiskan microplate spectrophotometer reader and expressed as percent cell viability in the culture medium after addition of a 10% Tween 80 solution.

After this analysis, the effect on viability of albendazole on both HaCat (human skin keratinocyte) and Vero (monkey kidney epithelial cell) cells was evaluated by MTT assay [44]. Firstly, the mammalian cells (5 × 10^4^/well) were allowed to adhere in 96-well tissue culture plates for 4 h at 37 °C in a 5% CO_2_ atmosphere. Non-adherent cells were removed by washes with sterile DMEM and the wells refilled with DMEM medium supplemented with 10% FBS. The cells were then incubated with increasing concentrations of albendazole (15.62 to 750 µM) and incubated for additional 24 h and 96 h at 37 °C in a 5% CO_2_ atmosphere. Subsequently, the culture medium was discharged, and the formation of formazan was measured by adding MTT (5 mg/mL in PBS, 25 µg/well) and incubating the wells for 3 h in the dark at 37 °C. Wells with cells receiving only the culture medium with or without 1% DMSO were also included as positive cellular growth controls. The plates were subsequently centrifuged at 500× *g* for 8 min, the supernatant was removed, the pellet was dissolved in 200 µL of DMSO, and the absorbance measured in an ELISA reader at 570 nm (SpectraMax Gemini 190, Molecular Devices, CA, USA). The 50% cytotoxicity inhibitory concentration (CC_50_) was determined by non-linear regression analysis.

The SI was calculated using the formula: SI = CC_50_ (µM)/MIC (µM). The higher the ratio obtained, the more selective the hit drug is against the pathogen.

### 2.7. Urease Production

To verify urease production, three strains: *Fonsecaea pedrosoi* (CFP 00791) *Cladophialophora carrionii* (CFP 00910), and *Exophiala heteromorpha* (ATCC^®^ 28180), corresponding to the three major chromoblastomycosis agents worldwide, were cultured on PDA for seven days and further cultured in Christensen urea broth [45] in the presence and absence of albendazole at a ½ MIC concentration. *Candida albicans* and *Cryptococcus neoformans* were used as negative and positive controls of the culture medium, respectively. In brief, fungal suspensions were adjusted to a concentration of 10^6^ cells/mL and used for inoculation. A volume of 500 μL of these suspensions were inoculated into 4.5 mL of Christensen urea broth and the tubes were then incubated under agitation (150 rpm) at 37 °C. At the end of seven and 12 days of incubation, the tubes were centrifuged, and a volume of 100 μL of the supernatant was transferred to a 96-well plate; this procedure was performed in triplicate. The absorbances of the samples were read on a SpectraMax plus 384 spectrophotometer (Molecular Devices, San José, CA, USA) at 559 nm, as previously described by Almeida-Paes et al. [46,47]. Results were analyzed statistically using the two-way ANOVA test with multiple comparisons. *p* values lower than 0.05 were considered significant.

### 2.8. Melanin Production

To verify melanin production in the presence of albendazole, the same three strains mentioned in the previous experiment were cultured in PDA media (positive DHN-melanin control), PDA supplemented with tricyclazole, an inhibitor of DHN melanin (melanin inhibition control), and PDA supplemented with ½ MIC albendazole concentration in order to verify if there is inhibition of melanin production induced by albendazole. At the end of the seven days of incubation at 37 °C, the plates were read visually. The aspect of the colonies supplemented with albendazole were compared to those of the positive melanin and inhibition melanin controls, to determine whether the color of the albendazole-supplemented cultures resembled the positive DHN-melanin or the melanin inhibition control. Pictures of the colonies were captured using a Canon EOS Rebel T7+ digital camera.

## 3. Results

### 3.1. NIH Clinical Collection Initial Screening

Among the 727 substances present in the NCC, 21 (2.9%) demonstrated antifungal activity against the CFP00791 *F. pedrosoi* isolate. Antifungal activity was observed in drugs with diverse primary uses, such as antihypertensive, anthelmintic, antidiabetic, antifungal, contraceptive, antihistamine, antineoplastic, antipsychotic, antibiotic, immunosuppressant, antidepressant, antiarrhythmic, and antiulcer agents (Table 1). In addition, the antifungal drug itraconazole, present in the collection, worked as a reference inhibition control that has inhibited 100% of fungal growth.

### 3.2. Applicability in the CBM Treatment

After analyzing the pharmacological data of the substances with anti-*F. pedrosoi* activity in the DrugBank platform, some substances were excluded from the present study: mesoridazine, tacrolimus, and flecainide were excluded due to serious adverse effects; olopatadine for having an administration route not recommended for CBM; medroxyprogesterone as it is contraindicated for the population most affected by CBM (male patients); 5-fluorocytosine, miconazole, econazole, and oxiconazole as they are already described as antifungal drugs; and 5- fluorouracil and carmofur as they belong to the same drugs class as 5-fluorocytosine, an already known antifungal drug with activity against some CBM agents. Therefore, 11 substances were excluded from the study, leaving 10 for investigation: linezolid, enalaprilat, miglitol, rabeprazole, cotinine, floxuridine, sertraline, albendazole, mebendazole, and thiabendazole.

### 3.3. Antifungal Activity

The MIC values of the 10 hit drugs from the NCC selected in the screening with the *Fonsecaea pedrosoi* (CFP 00791) strain were determined with other CBM agents. Linezolid, enalaprilat, miglitol, rabeprazole, and cotinine showed MIC values above the highest concentration tested (80 µM) for the different species that cause CBM. Floxuridine showed MIC values above 80 µM, except for the *Fonsecaea nubica* isolate (MIC = 0.3125 µM). Sertraline presented MIC values ranging between 10 µM and 40 µM, has systemic action, especially in the central nervous system, and would have a greater indication for cases of pheohyphomycosis. We then continued to compare the action of benzimidazoles, which presented MICs ranging from 0.625 to 20 µM. Regarding the determination of MFC, we observed that albendazole was fungicidal for *F. pedrosoi* and *F. monophora*, mebendazole was fungicidal for *C. carrionii, P. verrucosa,* and *F. pedrosoi*, while thiabendazole was fungicidal for *C. carrionii*, *F. pedrosoi*, *F. monophora,* and *F. nubica*. The MIC, MFC, and MFC/MIC values of the respective drugs are summarized in the Table 2.

### 3.4. Evaluation of Cytotoxicity and Determination of the Selectivity Index (SI) of Benzimidazoles

**Figure 1:**
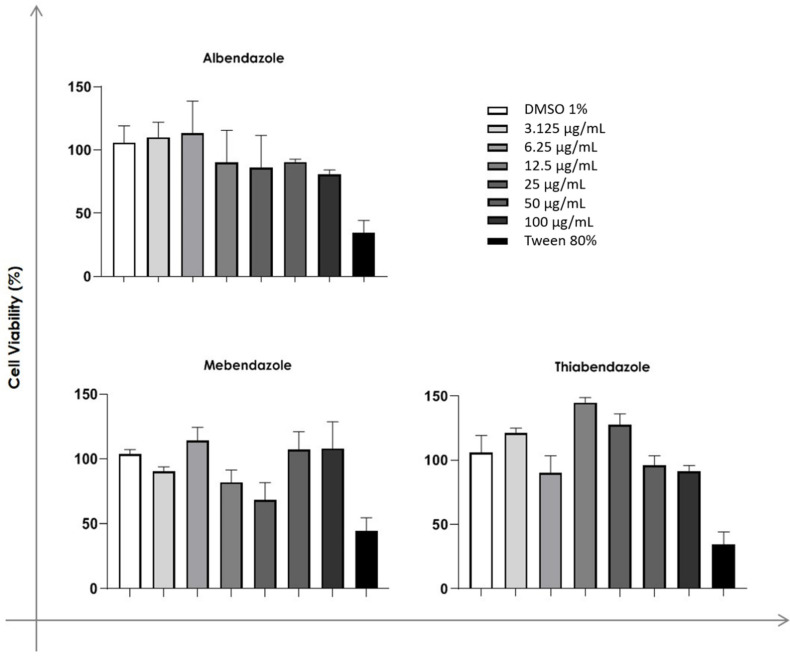
Concentration-dependent cytotoxic effects of albendazole, mebendazole, and thiabendazole on monkey kidney epithelial cells (VERO). Results are expressed as percentage of total cell viability (spectrophotometric readings) in the culture medium after addition of a 5 mg/mL MTT solution (positive response). The height of the histogram bar is the mean ± SEM of three independent experiments. MTT, 3-(4,5-dimethylthiazol-2-yl)-2,5-diphenyltetrazolium bromide.

The CC50 values for the benzimidazoles on the mammalian VERO cells (Figure 1) were found to be >100 μg/mL. This translates to approximately 377.35 μM for albendazole, 338.98 μM for mebendazole, and 497.51 μM for thiabendazole. After this initial analysis, the effect of albendazole on HaCat (human skin keratinocyte) and Vero (monkey kidney epithelial cell) cells viability was evaluated and the CC_50_ values were 85.1 µM for HaCat and 261.56 µM for Vero cells after 96 h. The data obtained pointed to a better selectivity of albendazole among the other benzimidazoles (Table 3).

### 3.5. Analysis of Synergism with Itraconazole and Terbinafine

Due to the higher selectivity of albendazole, its synergism with itraconazole and terbinafine against the CBM agents was investigated. The FICI was determined and is detailed in Table 4. All tested isolates showed indifferent interaction with combinations of albendazole with itraconazole and albendazole with terbinafine.

### 3.6. Virulence Factor Expression in the Presence of Albendazole

**Figure 2:**
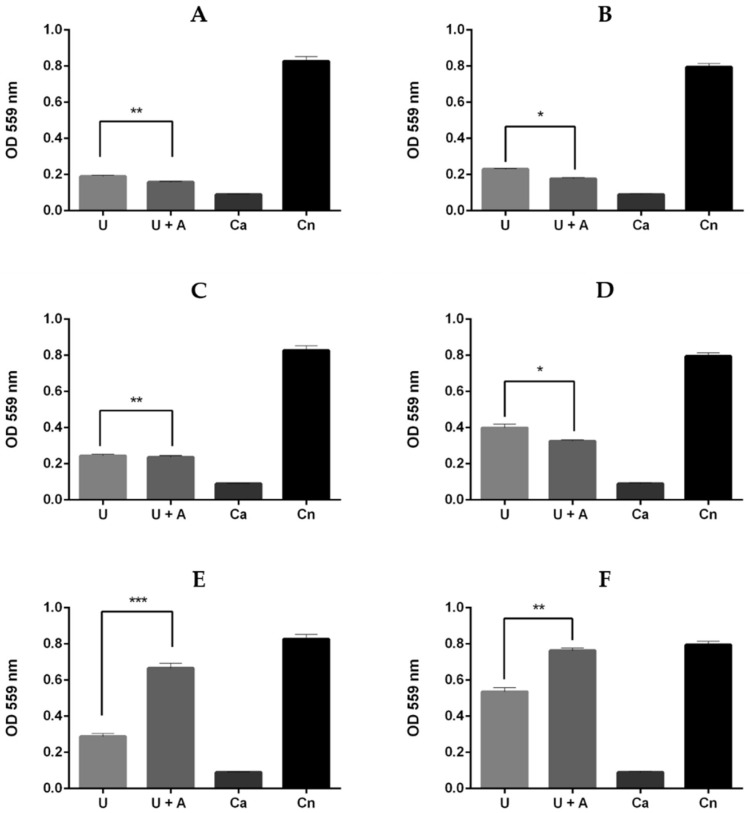
Urease production, inferred after optical density (OD) readings at 559 nm of three chromoblastomycosis agents at two different time points. The analyses showed different levels of statistical significance determined using two-way ANOVA represented by * (*p* < 0.05), ** (*p* < 0.01), and *** (*p* < 0.001). (**A**) *Cladophialophora carrionii*, 7 days; (**B**) *C. carrionii*, 12 days; (**C**) *Exophiala heteromorpha*, 7 days; (**D**) *E. heteromorpha*, 12 days; (**E**) *Fonsecaea pedrosoi*, 7 days; and (**F**) *F. pedrosoi*, 12 days. In all panels: U: chromoblastomycosis agent in the Christensen’s urea broth; U + A: chromoblastomycosis agent in the Christensen´s urea broth supplemented with ½ albendazole MIC; Ca: *Candida albicans* negative control; and Cn: *Cryptococcus neoformans* positive control.

Upon measuring the optical densities of the culture supernatants at 559 nm, we made several noteworthy observations. Firstly, in the *F. pedrosoi* strain, the presence of albendazole in the urea broth resulted in an increased secretion of urease. Conversely, in the *C. carrionii* and *E. heteromorpha* isolates, we observed a decrease in urease secretion (*p*-values = 0.0004, 0.0012, and 0.0041, respectively) (Figure 2). It is worth mentioning that these results remained consistent even after 12 days of incubation at 37 °C.

**Figure 3:**
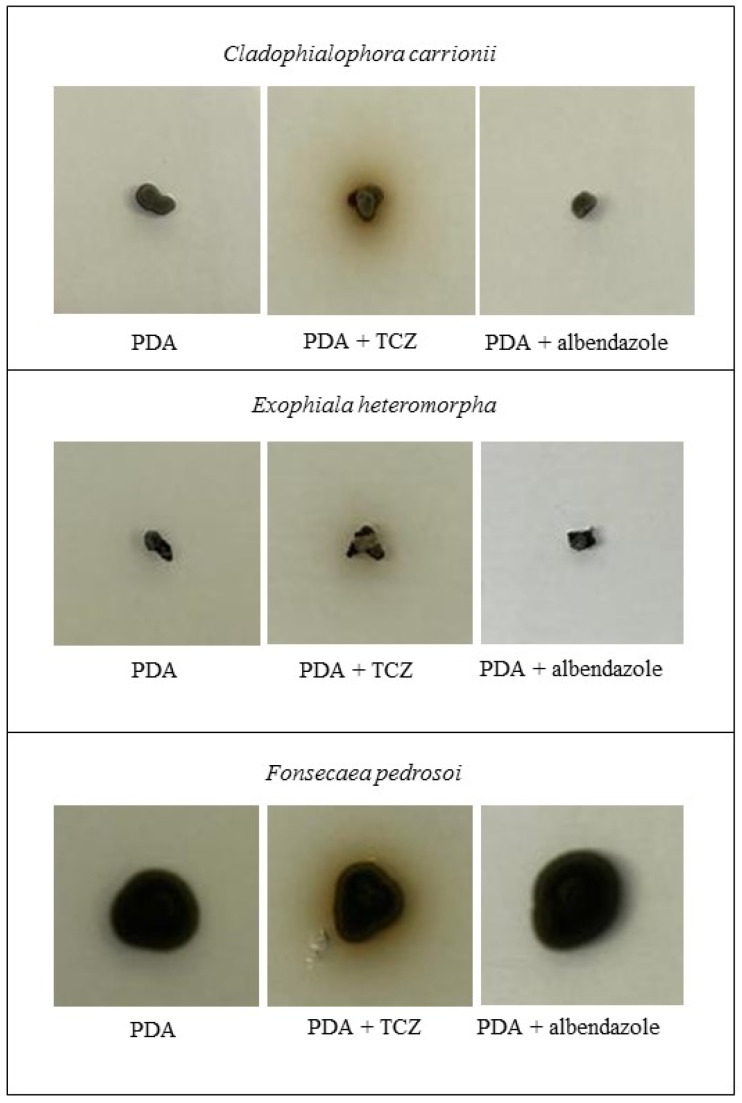
Melanin production on Potato Dextrose Agar (PDA) plates by three chromoblastomycosis agents at subinhibitory concentrations of albendazole compared to tricyclazole (TCZ), a DHN-melanin inhibitor.

Regarding melanin production, we did not observe a decrease in melanin production when the fungus was cultivated under albendazole subinhibitory concentrations. The presence of tricyclazole, a DHN melanin inhibitor, in the culture medium changes the color of colonies from dark green, gray or black (due to melanin) to reddish brown, which was observed in the controls. The color of albendazole supplemented cultures was similar to that observed for melanin controls, for all species tested (Figure 3).

## 4. Discussion

Drug repositioning is a strategy used to facilitate the treatment of several infectious diseases, especially tropical neglected diseases [21,22,23]. A collection of substances—NIH Clinical Collection—was used in this study to identify drugs with antifungal activity against several CBM agents, and therefore with repurposing potential for CBM. This collection has been previously tested on other fungi [35,48,49], and, as observed here, benzimidazoles were also found to have antifungal activity.

Our screening revealed 21 drugs with antifungal activity against *F. pedrosoi*, the main CBM agent. We proceeded with our investigation for 10 out of these 21 compounds. Mesoridazine, commercially known as Serentil, is an antipsychotic drug withdrawn from the market in 2005 for causing cardiac arrhythmia, so its use for months in patients with CBM would be discouraged. Olopatadine, also known as Patanol, is an antihistamine eye drop that is not indicated for the study, as CBM is not commonly associated with ocular lesions [50]. Tacrolimus is an immunosuppressive drug and its use is not indicated for fungal infections, since the cellular immune response plays an important role in the control of CBM [51,52]. Medroxyprogesterone is a contraceptive; it was excluded because CBM mainly affects males [53]. 5-fluorocytosine is a pyrimidine analogue antifungal drug that has already been used in the treatment of CBM [54,55,56]. Studies showed that *F. pedrosoi* has high MIC values against this drug [19], and in addition, this agent seems to develop resistance to fluorocytosine [55,57]; the same occur for 5-fluorouracil, as it is a metabolite of fluorocytosine in the fungal organism, and carmofur, which belongs to the same class as 5-fluorocytosine. Flecainide is used to treat cardiac arrhythmia [58], and therefore, was excluded from the study. Miconazole, econazole, and oxiconazole nitrates are already known topical antifungal drugs [59]. Sertraline is a drug with a systemic effect that acts on the central nervous system, making it interesting to investigate its use in cases of cerebral pheohyphomycosis, since CBM usually does not present with neurological dissemination [57]. Sertraline has antifungal activity against other fungi [60,61,62], but the MIC values against the eight CBM agents tested was higher than those previously published for other fungi.

Benzimidazole derivatives have been associated with the control of infectious diseases through antiviral, antifungal, antimicrobial, and antiprotozoal properties, but they also manifest anti-inflammatory, anticancer, antioxidant, anticoagulant, antidiabetic, and antihypertensive activities [63,64]. Some studies have demonstrated antifungal activity of benzimidazoles such as flubendazole [65], albendazole [66], and mebendazole [35] against *Cryptococcus neoformans*, fenbendazole [67] against *Cryptococcus gattii* and *C. neoformans*, albendazole against *Aspergillus* spp. [68], and, more recently, mebendazole against *Histoplasma capsulatum* [49]. Similarly, our findings demonstrated antifungal activity in a group of anthelmintic benzimidazoles against CBM agents, suggesting potential targets for the development of new broad-range antifungal drugs.

The anti-cryptococcal activity of benzimidazoles was demonstrated two decades ago [69], but the effects of these compounds on *Cryptococcus* morphology and biofilm formation had not been explored at that time. The study by Joffe et al., using the same drug library that we used here, found anti-cryptococcal activity on mebendazole. This drug showed antifungal activity against phagocytosed *C. neoformans*, profoundly affected cryptococcal biofilms, and caused marked morphological changes, including reduced capsular dimensions [35]. As CBM agents do not form a capsule and histological sections do not demonstrate fungal biofilms in parasitism, but only in isolated muriform bodies [2,70], we suggest that the mebendazole activities against *Cryptococcus* and CBM agents are different.

Albendazole, mebendazole, and thiabendazole showed in vitro antifungal activity against *F. pedrosoi* in the initial screening of the NCC library. In the past, thiabendazole has been used to treat CBM [57,71,72,73,74], however its use was discouraged due to relapses of the disease [75]. Among the benzimidazoles, albendazole was the one that presented the lowest MIC values, and consequently, the greatest selectivity against CBM agents. The antifungal activity of albendazole against *C. neoformans* is known [66] and different pathogenic species of *Aspergillus* [68]. Future clinical trials will be necessary to assess whether albendazole would also be associated with cases of CBM recurrence.

The study by Marslin et al. (2017) observed that this drug, in addition to the anthelmintic effect, has an anticancer property and has low toxicity to healthy cells. However, it is a drug of low gastrointestinal absorption (5%) and low oral bioavailability, as it has high crystallinity, low pharmaceutical processability, low solubility, and pH-dependence and precipitation of the drug when entering the intestine [76,77,78], which would make it a candidate only for topical treatment in early or mild forms of CBM. On the other hand, in 2021, the study by Sutar et al. demonstrated that the difficulty in repurposing benzimidazoles as a therapy for cryptococcal meningitis could be solved by transforming them into partially amorphous low-melting ionic liquids with a simple metathesis reaction using docusate amphiphilic sodium as a counterion. Thus, the development of docusate-based ionic liquids represents an effective approach to improve the physicochemical properties and potency of anthelmintic benzimidazoles to facilitate their repositioning and preclinical development for use as an antifungal drug [66], potentially including CBM agents.

Regarding virulence factors, it is known that CBM agents produce melanin, which is important in drug resistance [79,80], and with possible action in inducing humoral and cellular responses of the host [81]. Another important virulence factor is the secretion of urease, a metalloenzyme that catalyzes the hydrolysis of urea to ammonia and carbon dioxide [82,83,84]. We observed that albendazole did not interfere with the melanization of the tested isolates. Fernandes et al. found that the dematiaceous fungus *Alternaria infectoria* activates DHN-melanin synthesis in response to certain antifungal drugs, possibly as a protective mechanism against these drugs [85], which was not observed with the tested CBM agents in the presence of albendazole in the current study. Regarding urease production, *C. carrionii* and *E. heteromopha* showed a decrease in urease production in the presence of albendazole, which also occurs with *C. neoformans* in the presence of cyclosporine [47] and propolis [86], two substances with inhibitory activity against this pathogenic yeast. In contrast, *F. pedrosoi* showed an increase in this enzyme in the presence of the drug. These findings highlight the differential responses of the tested fungal isolates to the presence of albendazole in the urea broth. Further investigation into the underlying mechanisms driving these variations in urease secretion may provide valuable insights into the interaction between the drug and different fungal species. It is worth mentioning that these results remained consistent even after twelve days of incubation at 37 °C. This suggests that the observed effects on urease secretion were sustained over time. Also, additional experiments and analyses were performed to ensure the accuracy and reliability of these results.

## 5. Conclusions

In conclusion, benzimidazoles have repositioning potential for the treatment of CBM. All were fungicidal against *F. pedrosoi*, the main CBM agent worldwide, and against several other agents of this mycosis. Among the benzimidazoles with activity against the dematiaceous fungi herein studied, albendazole is suggested as the best option for drug repurposing, as it showed the highest selectivity index with all CBM agents tested. This drug can be used in the future in topical formulations, as it is currently recommended for other diseases [87], or, after modifications that improve its bioavailability, in oral or injectable formulations for this neglected mycosis. Future clinical trials are necessary to address this issue.

## Figures and Tables

**Table 1:** Drugs from the NIH Clinical Collection presenting antifungal activity at 10 µM in the screening against the CFP00791 *Fonsecaea pedrosoi* isolate.

Drug	Primary Use	% Growth Inhibition	Formulation	Class
Thiabendazole	Anthelmintic	82	Oral Topic	Benzimidazoles
Miglitol	Antidiabetic	90	Oral	-
Medroxyprogesterone	Contraceptive	91	Oral Injectable	-
Olopatadine (Patanol)	Antihistamine	85	Eye drops	-
Carmofur	Antineoplastic	100	Oral	Pyrimidine analogue
Mesoridazine (Serentil)	Antipsychotic	86	Oral Intravenous	Phenothiazines
Linezolid	Antibiotic	87	Oral Intravenous	Oxazolidinones
Tacrolimus	Immunosuppressant	89	Oral	Calcineurin inhibitors
Enalaprilat	Antihypertensive	67	Intravenous	Carboxylic acids
Albendazole	Anti-helminthic and anti-protozoal	98	Oral	Benzimidazoles
Oxiconazole (nitrate)	Antifungal	91	Topic	Imidazole
Mebendazole	Anthelmintic	83	Oral	Benzimidazoles
Floxuridine	Oncology drug	86	Injectable	Pyrimidine analogue
Sertraline	Antidepressant	90	Oral	Tetralin
Flecainide Acetate	Treatment of cardiac arrhythmia	95	Oral	Antiarrhythmics
Cotinine (nicotine)	Alkaloid found in tobacco, predominant metabolite of nicotine	71	Nasal spray Inhalant Transdermal Oral	-
Econazole nitrate	Antifungal	94	Topic	Imidazole
Miconazole nitrate	Antifungal	99	Topic Vaginal cream	Imidazole
Rabeprazole	Antiulcer	61	Oral	Benzimidazoles (antisecretory)
5-Fluorouracil	Antineoplastic	85	Topic Intravenous	Halopyrimidine
5-Fluorocytosine	Antifungal	68	Oral	Halopyrimidine

**Table 2:** Determination of the minimum inhibitory concentration (MIC) and minimum fungicidal concentration (MFC) (μM) of three benzimidazoles against eight chromoblastomycosis agents.

Species	Albendazole			Mebendazole			Thiabendazole		
	MIC	MFC	MFC:MIC Ratio	MIC	MFC	MFC:MIC Ratio	MIC	MFC	MFC:MIC Ratio
*C. carrionii*	2.5	20	8	2.5	2.5	1	5	5	1
*P. verrucosa*	0.625	40	64	5	10	2	5	20	4
*E. dermatitidis*	2.5	>80	>32	20	>80	>4	10	80	8
*E. heteromorpha*	2.5	80	32	5	>80	>16	10	80	8
*F. pedrosoi*	1.25	1.25	1	5	5	1	5	5	1
*F. monophora*	0.625	0.625	1	2.5	80	32	5	5	1
*F. nubica*	0.625	40	64	5	>80	>16	5	5	1
*R. similis*	1.25	>80	>64	5	>80	>16	10	40	4

**Table 3:** Selectivity index (SI) of three benzimidazoles for eight species that cause chromoblastomycosis.

Species	Selectivity Index (SI)				
	Thiabendazole	Mebendazole	Albendazole	Albendazole	
	Vero 24 h	Vero 24 h	Vero 24 h	Vero 96 h	HaCat 96 h
*C. carrionii*	99.50	135.59	150.94	104.62	34.04
*P. verrucosa*	99.50	67.79	603.76	418.49	136.16
*E. dermatitidis*	49.75	16.94	150.94	104.62	34.04
*E. heteromorpha*	49.75	67.79	150.94	104.62	34.04
*F. pedrosoi*	99.50	67.79	301.88	209.24	68.08
*F. monophora*	99.50	135.59	603.76	418.49	136.16
*F. nubica*	99.50	67.79	603.76	418.49	136.16
*R. similis*	49.75	67.79	301.88	209.24	68.08

Vero = monkey kidney epithelial cells; HaCat = human skin keratinocyte.

**Table 4:** Fractional inhibitory concentration index (FICI) of albendazole with terbinafine and itraconazole against eight species that cause chromoblastomycosis.

Species	FICI Albendazole + Itraconazole	FICI Albendazole + Terbinafine
*C. carrionii*	1.06	1.06
*P. verrucosa*	1.125	2.12
*E. dermatitidis*	1.06	1.06
*E. heteromorpha*	1.03	1.06
*F. pedrosoi*	1	1.12
*F. monophora*	1.50	1.12
*F. nubica*	1.25	1.12
*R. similis*	0.62	2

## Data Availability

The data presented in this study are available within the article.

## Supplementary Materials

The following supporting information can be downloaded at: https://www.mdpi.com/article/10.3390/jof9070753/s1, Figure S1: Layout of chequerboard plates used in synergism assays with the concentrations of drugs tested in the different wells. (A) Combination albendazole and itraconazole; (B) combination albendazole and terbinafine.
