# Supplementary material for: Repurposing Benzimidazoles against Causative Agents of Chromoblastomycosis: Albendazole Has Superior In Vitro Activity Than Mebendazole and Thiabendazole
Source: J Fungi (Basel). 2023 Jul 16;9(7):753. doi: 10.3390/jof9070753 (PMC10381309; doi:10.3390/jof9070753)
Supplement: Supplementary file 1 [file jof-09-00753-s001.zip › jof-2356660-supplementary.pdf]

(A) albendazole (ALB) x itraconazole (ITZ) (µM)

	1	2	3	4	5	6	7	8	9	10	11	12
A	NC	ALB 64.0 ITZ 4.0	ALB 32.0 ITZ 4.0	ALB 16.0 ITZ 4.0	ALB 8.0 ITZ 4.0	ALB 4.0 ITZ 4.0	ALB 2.0 ITZ 4.0	ALB 1.0 ITZ 4.0	ALB 0.5 ITZ 4.0	ALB 0.25 ITZ 4.0	ALB 0.125 ITZ 4.0	**ITZ 4.0**
B	NC	ALB 64.0 ITZ 2.0	ALB 32.0 ITZ 2.0	ALB 16.0 ITZ 2.0	ALB 8.0 ITZ 2.0	ALB 4.0 ITZ 2.0	ALB 2.0 ITZ 2.0	ALB 1.0 ITZ 2.0	ALB 0.5 ITZ 2.0	ALB 0.25 ITZ 2.0	ALB 0.125 ITZ 2.0	**ITZ 2.0**
C	NC	ALB 64.0 ITZ 1.0	ALB 32.0 ITZ 1.0	ALB 16.0 ITZ 1.0	ALB 8.0 ITZ 1.0	ALB 4.0 ITZ 1.0	ALB 2.0 ITZ 1.0	ALB 1.0 ITZ 1.0	ALB 0.5 ITZ 1.0	ALB 0.25 ITZ 1.0	ALB 0.125 ITZ 1.0	**ITZ 1.0**
D	NC	ALB 64.0 ITZ 0.5	ALB 32.0 ITZ 0.5	ALB 16.0 ITZ 0.5	ALB 8.0 ITZ 0.5	ALB 4.0 ITZ 0.5	ALB 2.0 ITZ 0.5	ALB 1.0 ITZ 0.5	ALB 0.5 ITZ 0.5	ALB 0.25 ITZ 0.5	ALB 0.125 ITZ 0.5	**ITZ 0.5**
E	NC	ALB 64.0 ITZ 0.25	ALB 32.0 ITZ 0.25	ALB 16.0 ITZ 0.25	ALB 8.0 ITZ 0.25	ALB 4.0 ITZ 0.25	ALB 2.0 ITZ 0.25	ALB 1.0 ITZ 0.25	ALB 0.5 ITZ 0.25	ALB 0.25 ITZ 0.25	ALB 0.125 ITZ 0.25	**ITZ 0.25**
F	NC	ALB 64.0 ITZ 0.125	ALB 32.0 ITZ 0.125	ALB 16.0 ITZ 0.125	ALB 8.0 ITZ 0.125	ALB 4.0 ITZ 0.125	ALB 2.0 ITZ 0.125	ALB 1.0 ITZ 0.125	ALB 0.5 ITZ 0.125	ALB 0.25 ITZ 0.125	ALB 0.125 ITZ 0.125	**ITZ 0.125**
G	NC	ALB 64.0 ITZ 0.0625	ALB 32.0 ITZ 0.0625	ALB 16.0 ITZ 0.0625	ALB 8.0 ITZ 0.0625	ALB 4.0 ITZ 0.0625	ALB 2.0 ITZ 0.0625	ALB 1.0 ITZ 0.0625	ALB 0.5 ITZ 0.0625	ALB 0.25 ITZ 0.0625	ALB 0.125 ITZ 0.0625	**ITZ 0.0625**
H	NC	**ALB 64.0**	**ALB 32.0**	**ALB 16.0**	**ALB 8.0**	**ALB 4.0**	**ALB 2.0**	**ALB 1.0**	**ALB 0.5**	**ALB 0.25**	**ALB 0.125**	**PC**

(B) albendazole (ALB) x terbinafine (TRB) (µM)

	1	2	3	4	5	6	7	8	9	10	11	12
A	NC	ALB 64.0 TRB 4.0	ALB 32.0 TRB 4.0	ALB 16.0 TRB 4.0	ALB 8.0 TRB 4.0	ALB 4.0 TRB 4.0	ALB 2.0 TRB 4.0	ALB 1.0 TRB 4.0	ALB 0.5 TRB 4.0	ALB 0.25 TRB 4.0	ALB 0.125 TRB 4.0	**TRB 4.0**
B	NC	ALB 64.0 TRB 2.0	ALB 32.0 TRB 2.0	ALB 16.0 TRB 2.0	ALB 8.0 TRB 2.0	ALB 4.0 TRB 2.0	ALB 2.0 TRB 2.0	ALB 1.0 TRB 2.0	ALB 0.5 TRB 2.0	ALB 0.25 TRB 2.0	ALB 0.125 TRB 2.0	**TRB 2.0**
C	NC	ALB 64.0 TRB 1.0	ALB 32.0 TRB 1.0	ALB 16.0 TRB 1.0	ALB 8.0 TRB 1.0	ALB 4.0 TRB 1.0	ALB 2.0 TRB 1.0	ALB 1.0 TRB 1.0	ALB 0.5 TRB 1.0	ALB 0.25 TRB 1.0	ALB 0.125 TRB 1.0	**TRB 1.0**
D	NC	ALB 64.0 TRB 0.5	ALB 32.0 TRB 0.5	ALB 16.0 TRB 0.5	ALB 8.0 TRB 0.5	ALB 4.0 TRB 0.5	ALB 2.0 TRB 0.5	ALB 1.0 TRB 0.5	ALB 0.5 TRB 0.5	ALB 0.25 TRB 0.5	ALB 0.125 TRB 0.5	**TRB 0.5**
E	NC	ALB 64.0 TRB 0.25	ALB 32.0 TRB 0.25	ALB 16.0 TRB 0.25	ALB 8.0 TRB 0.25	ALB 4.0 TRB 0.25	ALB 2.0 TRB 0.25	ALB 1.0 TRB 0.25	ALB 0.5 TRB 0.25	ALB 0.25 TRB 0.25	ALB 0.125 TRB 0.25	**TRB 0.25**
F	NC	ALB 64.0 TRB 0.125	ALB 32.0 TRB 0.125	ALB 16.0 TRB 0.125	ALB 8.0 TRB 0.125	ALB 4.0 TRB 0.125	ALB 2.0 TRB 0.125	ALB 1.0 TRB 0.125	ALB 0.5 TRB 0.125	ALB 0.25 TRB 0.125	ALB 0.125 TRB 0.125	**TRB 0.125**
G	NC	ALB 64.0 TRB 0.0625	ALB 32.0 TRB 0.0625	ALB 16.0 TRB 0.0625	ALB 8.0 TRB 0.0625	ALB 4.0 TRB 0.0625	ALB 2.0 TRB 0.0625	ALB 1.0 TRB 0.0625	ALB 0.5 TRB 0.0625	ALB 0.25 TRB 0.0625	ALB 0.125 TRB 0.0625	**TRB 0.0625**
H	NC	**ALB 64.0**	**ALB 32.0**	**ALB 16.0**	**ALB 8.0**	**ALB 4.0**	**ALB 2.0**	**ALB 1.0**	**ALB 0.5**	**ALB 0.25**	**ALB 0.125**	**PC**

NC: Negative control (culture medium only)
PC: Positive control (culture medium and fungal inoculum, no drugs)

Figure S1. Layout of chequerboard plates used in synergism assays with the concentrations of drugs tested in the different wells. (**A**) Combination albendazole and itraconazole; (**B**) combination albendazole and terbinafine.
